# Economic Burden and Medical Insurance Impact of the Different Dialysis for End-stage Renal Diseases

**Published:** 2018-11

**Authors:** Dan GAO, Sanhui JING, Jian WU, Ge WU

**Affiliations:** 1. Dept. of Nephrology, First Affiliated Hospital of Zhengzhou University, Zhengzhou, Henan, China; 2. College of Public Health, Zhengzhou University, Zhengzhou, Henan, China

**Keywords:** End stage renal disease, Hemodialysis, Peritoneal dialysis, Medical insurance

## Abstract

**Background:**

Dialysis costs was a heavy burden in End Stage Renal Disease (ESRD) patients. In China, the two major medical insurance systems are the New Cooperative Medical Scheme (NCMS) for rural residents and the Urban Employees’ Medical Insurance (UEMI) for urban patients. This study compared the economic burden of ESRD patients under different dialysis methods and the impact of the medical insurance system on it.

**Methods:**

Overall, 156 ESRD patients were enrolled at the Department of Nephrology in the First Affiliated Hospital of Zhengzhou University, Zhengzhou, China between Jan 2013 and Jan 2014. They were divided into hemodialysis group (HD group, n=84) and peritoneal dialysis group (PD group, n=72). The data, such as the patient’s basic information, total expenses and self-paid expenses in the early stage of dialysis and 1-year treatment, and medical insurance type, were separately collected.

**Results:**

The early-stage average total expenses and self-paid expenses in the PD group were higher than those in the HD group (*P*<0.01). The average total expenses and self-paid expenses in the PD group were lower than those in the HD group (*P*<0.01). Whichever dialysis method was used, the self-paid expense percentage for the NCMS patients and was higher than UEMI patients.

**Conclusion:**

In terms of the long-term dialysis treatment for ESRD patients, the better choice was PD judging by the treatment expenses. Meanwhile, different medical insurance types had significant economic burden impacts on dialysis patients.

## Introduction

End Stage Renal Disease (ESRD) is one of the serious diseases that threaten human health in the world, and its incidence shows an increasing trend year by year. According to the renal disease data system in the US, the ESRD patients increase at the rate of 1.9%–2.3% (1). The prevalence rate of Chronic Kidney Disease (CKD) in Mainland China is up to 10.8% (2). Of the 120 million CKD patients, about 2% develop into ESRD patients. About 2–3 million people need to receive the long-term renal replacement therapy (3), namely hemodialysis, peritoneal dialysis and renal transplantation, etc., in order to ensure the basic life quality. Specifically, hemodialysis and peritoneal dialysis are the major treatment methods for ESRD in China.

However, with the rapid growth of the dialysis population in recent years, the dialysis costs have kept increasing in various countries, and the ESRD patients have become a heavy burden on society and families. With the constant improvement of the medical insurance system in China, the economic burden of dialysis patients was relieved to vary degrees. But which dialysis method has better cost benefits and can reduce the payment pressure of the medical healthcare insurance fund while relieving the burden on patients has become a hot and difficult issue for the professional fields and medical insurance authorities in China.

This study chose the regular peritoneal dialysis patients and hemodialysis patients of the First Affiliated Hospital of Zhengzhou University, collected such data as patients’ medical insurance type, differences of total expenses in the early stage of dialysis, 1-year total treatment expenses, and self-paid expenses, compared and analyzed the differences between economic burden of different dialysis methods and different medical insurance policies on their compensation amount, and provided empirical basis for relieving the economic burden of dialysis treatment on ESRD patients, for choosing the dialysis method and for perfecting the medical insurance policy.

## Materials and Methods

The ESRD patients who received peritoneal dialysis and hemodialysis at the First Affiliated Hospital of Zhengzhou University for the first time in Henan Province from Jan 2013 to Jan 2014 were chosen. They were filtered and chosen one by one according to the inclusion and exclusion criteria, and 156 patients were chosen. Specifically, there were 72 patients in the peritoneal dialysis group, and 84 patients in the hemodialysis group, including 91 males and 65 females. Those patients who met the inclusion and exclusion conditions were contacted by phone and followed up face to face, and were informed of the study issues and the requirements to cooperate with the follow-up, and their opinions were sought for. The questionnaire survey was conducted to collect data.

This study was approved by the Ethical Committee of the First Affiliated Hospital of Zhengzhou University and all the participants provided a written informed consent.

We defined the inclusion criteria as follows: 1) Aged 18–75; 2) Compliance with the ESRD diagnosis according to the 2012 KDIGO guide; 3) They must be the patients who establish the PD or HD channels at our hospital for the first time; 4) They must have received regular dialysis (namely HD 3 times/week, PD CAPD) for at least 1 year, and have been followed up on a regular basis; 5) They must have a clear mind, normal memory, have no serious complications concerning their hearts, lungs, brains, and livers, etc., and agree to cooperate in this survey.

The exclusion criteria as follows: 1) Aged <18 or >75; 2) Dialysis time <1 year or dialysis is not regular; 3) People with mental disorders, disturbance of consciousness, or abnormal communications or serious complications, who cannot cooperate in the survey;

The collection of data on general conditions includes the name, gender, age, occupation, medical Insurance type, dialysis way, total cost of dialysis in the early stage, total cost of 1-year treatment, etc. The pre-dialysis costs refer to the costs incurred in the preparatory stage of dialysis, which mainly included the diagnosis fee, treatment fee, medicine fees, general medical service fee, etc. The 1-year total costs mainly include the dialysis treatment cost, complication drug cost, and re-examination cost. The medical insurance types include the New Cooperative Medical Scheme (NCMS) for rural residents and the Urban Employees’ Medical Insurance (UEMI) for urban patients.

For data collection, the retrospective analysis method was used. Overall, 156 patients who received PD and HD at the First Affiliated Hospital of Zhengzhou University for the first time in Henan Province from Jan 2013 to Jan 2014 were chosen, including 72 patients in the PD group and 84 patients in the HD group. All the data should be input by double persons in double copies using Excel 2007 to establish the database for data management. For statistical analysis, the SPSS 19.0 software (Chicago, IL, USA) was used for data analysis. The measurement data were expressed with mean ± standard deviation. The abnormal distribution data were turned into normal distribution data via logarithmic transformation, then were analyzed. The comparison between the two groups employed the t-test. The differences had statistical significance if *P*<0.05.

## Results

### Basic information on objects of study

This study included 156 ESRD patients with regular dialysis, including 31 patients aged above 60 (19.9%), 103 patients aged 30–60 (66.0%), and 22 patients aged below 30 (14.1%) (Table 1).

**Table 1:** Age structure of dialysis patients

***Age(yr)***	***n***	***Percentage (%)***
Below 30	22	14.1
30–60	103	66.0
Above 60	31	19.9

### Medical insurance type for dialysis patients

Of the subjects of this study, 89 people (accounting for 57.05%) participated in the NCMS, 24.36% were covered by the UEMI (Table 2).

**Table 2:** Medical security for dialysis patients

***Medical insurance type***	***PD patients Number***	***HD patients Number***	***Total patients***	***Percentage (%)***
UEMI	33	5	38	24.36
NCMS	35	54	89	57.05
Others	16	13	29	18.59

### Costs of different dialysis methods

#### Total costs before dialysis

The statistical result of pre-dialysis costs showed that the total cost of the PD group was about $5,810, and the total cost of the HD group was about $3,470. The total cost of the PD group before dialysis was higher than that of the HD group (*P*<0.01 in both cases), which had statistical significance (Table 3).

**Table 3:** Pre-dialysis costs of different dialysis methods

***Cost***	***PD group ($)***	***HD group ($)***	***t***	***P***
Total cost	5810±2030	3470±1820	6.26	<0.01
Self-paid cost	3960±1710	2020±1080	6.99	<0.01

#### Total cost for 1-year dialysis

The statistical result of the 1-year dialysis showed that the total cost of the PD group was $14,560, and the total cost of the HD group was $18250. The total cost of the PD group was lower than that of the HD group (*P*<0.01 in both cases), which had statistical significance (Table 4).

**Table 4:** Total 1-year treatment cost for patients using different dialysis ways

***Cost***	***PD group ($)***	***HD group ($)***	***t***	***P***
Total 1-year cost	14560±6010	18250±7610	2.65	0.009
Self-paid cost	5520±3000	7400±4360	2.49	0.014

### Patient expenses under different medical insurance policies

#### Expenses under different medical insurance policies for patients in PD group

Comparison of the expenses for PD patients under different medical insurance policies showed that for the NCMS patients, the early-stage total expenses and self-paid expenses were $5,990 and $3,520 respectively, the 1-year total expenses was $13,580, the self-paid expenses were $5,330, all lower than those of UEMI patients, but the self-paid expense percentage for the NCMS patients was higher than UEMI patients (Table 5).

**Table 5:** Expenses under different medical insurance policies for patients in PD group

***Medical insurance type***	***Early stage expense ($)***		***Percentage of self-paid fee %***	***1-year expense ($)***		***Percentage of self-paid fee %***
	***Total expense***	***Self-paid***		***Total expense***	***Self-paid***	
UEMI	2650±1950	1240±940	45440±13670	16940±8070	5960±2870	36060±9250
NCMS	5990±1920	3520±950	59870±6940	13058±4730	5330±3080	38980±16830

#### Expenses under different medical insurance policies for patients in HD group

In terms of the total expenses for HD patients, the NCMS patients had lower expense levels than UEMI patients in terms of both early-stage total expenses and 1-year total expenses. However, judging by the self-paid expenses, the self-paid expense percentage for the NCMS patients was higher than that of UEMI patients (Table 6).

**Table 6:** Expenses under different medical insurance policies for patients in HD group

***Medical insurance type***	***Early stage expense ($)***		***Percentage of self-paid fee %***	***1-year expense ($)***		***Percentage of self-paid fee %***
	***Total expense***	***Self-paid***		***Total expense***	***Self-paid***	
UEMI	4820±2210	1700±1280	32.79±13.35	20520±7060	7410±4410	37.13±15.68
NCMS	3150±1600	1860±790	62.42±10.95	14190±7090	7380±4410	50.55±12.13

## Discussion

Hemodialysis and peritoneal dialysis are the commonly used kidney replacement methods for ESRD patients. Hemodialysis can effectively clear metabolites and excessive moisture in the body, maintain electrolytes and acid-base balance and is the first choice for most of the ESRD patients. Peritoneal dialysis can protect the residual renal function and is portable and has been widely used in recent years. The patients using PD have better perception function and quality of life than the patients using HD. In Hong Kong, China, the ESRD patients tend to choose PD, accounting for 72.9% of the total dialysis population (4). However, whichever dialysis method requires massive manpower and material resources and causes a heavy burden on a country and individual. The ESRD patients are mainly aged 30–60 and are the major labor of their families and society, as well as the important factors that affect the social and economic development (Table 1).

Concerning the dialysis cost, the cost of the PD group patients before dialysis is higher than that of the HD group patients by about $2,260 (Table 3). The major possible causes include 1) Different ways of anesthesia: The peritoneal dialysis operation is a general anaesthesia operation, and the hemodialysis channel establishment is a local anesthesia operation. The cost for general anaesthesia operation is about $205, while the cost for local anesthesia is only $13. 2) Different operation costs: The cost of the traditional laparotomy PD operation is about $161, the cost of laparoscopic PD catheterization is about $291, the cost of establishment of the HD long-term pipe is $969, the cost of arteriovenous fistula plasty is $242, which shows that the PD operation cost is less than that of HD (The above costs are based on the charging standard of the First Affiliated Hospital of Zhengzhou University). 3) Different preoperative preparations: PD has high requirements for a patient’s conditions, and the cost of preoperative preparations is high. 4) Different length of hospitalization: The PD group patients need to receive formal training seven days after pipe establishment. The HD group patients may be discharged from hospital after 1 day of observation after channel establishment. Therefore, the PD group patients are hospitalized longer than the HD group patients and require higher costs.

Table 4 shows that in terms of the total treatment costs for the patients of the two groups 1 year later, the PD group is about $14560, the HD group is about $18250 and is equivalent to the cost as shown by the national survey. The possible causes may include 1) Different dialysis costs: At municipal-level hospitals, the cost of hemodialysis each time is about $57–80, while for the peritoneal dialysis patients, the cost of each bag of dialysate is about 6$. In the case of regular and full dialysis, the cost of the HD patients is certainly higher than that of the PD patients. 2) Different numbers of hospitalizations: PD may be conducted at home after channel establishment, while HD must be conducted at hospitals 3 times every week on average. Therefore, PD may save transport and hospitalization costs, etc. 3) Drugs: For HD patients, blood drainage out of the body may result in unstable hemodynamics and chronic blood loss, but for PD, this situation does not occur. Therefore, PD patients use fewer drugs such as antihypertensive drugs and erythropoietin. Therefore, judging by the 1-year treatment cost, the PD group families have a lighter economic burden than the HD group families.

Tables 5 and 6 show that judging by the expense levels, the total dialysis expenses and the self-paid expense for the NCMS patients were lower than UEMI patients, but the self-paid expense percentage for the NCMS patients was higher. This is probably because the basic income of the rural residents in Henan Province is far lower than that of urban residents, some patients might stop using the auxiliary drugs, such as drugs for anemia correction, electrolyte disturbance correction, and blood pressure control, or bought cheaper drugs, or failed to follow the doctor’s advice and pay return visits, which made the expenses of such patients low. However, high medical expenses still resulted in a large number of “illness-related poverty” phenomena (5). Judging by the reimbursement proportion, the self-paid expense percentage for the NCMS patients was about 50%–60% and was higher than that of other medical insurance patients.

The reasons may include: 1) The reimbursement proportion for the NCMS patients as defined by the national medical insurance policy is obviously lower than that defined by the provincial, and municipal and employees’ medical insurance, which is unfair to some extent. 2) Restriction of the hierarchical diagnosis and treatment policy: As the ESRD patients have many serious complications, some of them need to be transferred to provincial hospitals for treatment, but the hierarchical diagnosis and treatment system stipulates that skip-level treatment will decrease a patient’s reimbursement proportion.

## Conclusion

Though the PD cost in the preparatory stage before dialysis is higher than the HD cost for ESRD patients, the 1-year treatment cost is obviously lower in the PD group than that in the HD group. By comparison, the PD patients have lighter family financial burden than the HD patients. Medical insurance organizations increase the reimbursement proportion, formulate the reimbursement policy which drives patients to voluntarily choose PD, so as to further ease patients’ economic burden and increase ESRD patients’ life quality. Meanwhile further cost-effectiveness analysis of peritoneal dialysis and hemodialysis be carried out to provide decision-makers with more decision basis and suggestions.

## Ethical considerations

Ethical issues (Including plagiarism, informed consent, misconduct, data fabrication and/or falsification, double publication and/or submission, redundancy, etc.) have been completely observed by the authors.
